# Neonatal Sacrococcygeal Mass: From Lipoma to Teratoma

**DOI:** 10.1002/ccr3.72475

**Published:** 2026-04-02

**Authors:** Delia Argüelles Balas, Rosa María Zubimendi Pérez, Carolina González González, Rafael Arroyo Crespo, Juan López Carnero

**Affiliations:** ^1^ Neonatology Unit, Paediatrics Department Hospital Universitario Infanta Leonor Madrid España; ^2^ Medical Oncology Department Hospital Universitario Infanta Sofía Madrid Spain

**Keywords:** lipoma, newborn infant, Sacrococcygeal region, Teratoma

## Abstract

Sacrococcygeal masses in neonates that appear benign, such as presumed lipomas, may conceal germ cell tumors. Careful clinical evaluation, functional anorectal assessment, and magnetic resonance imaging are essential to differentiate benign lesions from sacrococcygeal teratomas and guide appropriate surgical management and follow‐up.

We present the case of a full‐term neonate with no relevant medical history who presented with a soft, mobile, non‐tender mass in the sacrococcygeal region (Figure 1). As part of the work‐up to exclude associated anomalies, cranial, abdominal, and spinal canal ultrasound scans, as well as an echocardiogram, were performed, all of which were unremarkable. Spontaneous passage of meconium and normal bowel transit supported the initial suspicion of a congenital lipoma. Other differential diagnoses of neonatal sacrococcygeal masses include meningocele, lipomyelomeningocele, vascular malformations, and epidermoid or dermoid cysts, which were considered but were unlikely given the normal spinal ultrasound findings.

**FIGURE 1:**
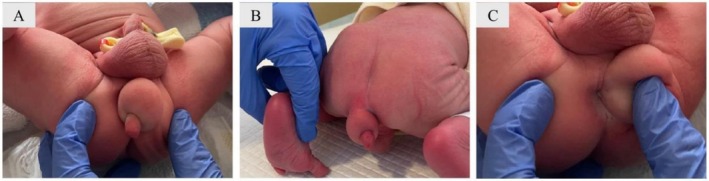
(A, B) A mass is observed in the sacrococcygeal region, to the left of the midline. It measures approximately 3–4 cm in diameter and has a soft, mobile consistency. An additional soft structure is seen attached at its lower right aspect. In image C, the presence of an anus is confirmed.

At 29 days of life, the patient was evaluated by pediatric surgery in an outpatient setting. Functional assessment with electrostimulation and anal calibration identified mild anal stenosis. The anal canal allowed a Hegar dilator size 8, and progressive dilatation was performed up to Hegar size 9 mm. Pelvic magnetic resonance imaging (MRI) demonstrated a fatty mass with internal solid nodules, findings suggestive of an Altman type I sacrococcygeal teratoma (Figure 2). Although alpha‐fetoprotein (AFP) levels are physiologically elevated in neonates, the measured AFP level was 126 μg/L (reference range 0.89–8.78 μg/L), supporting the suspicion of a germ cell tumor. Complete surgical resection, including coccygectomy was performed to reduce the risk of recurrence, and histopathological examination confirmed the diagnosis of a mature teratoma. The resected specimen measured 5.2 × 3.7 × 3.5 cm and weighed 37.9 g.

**FIGURE 2:**
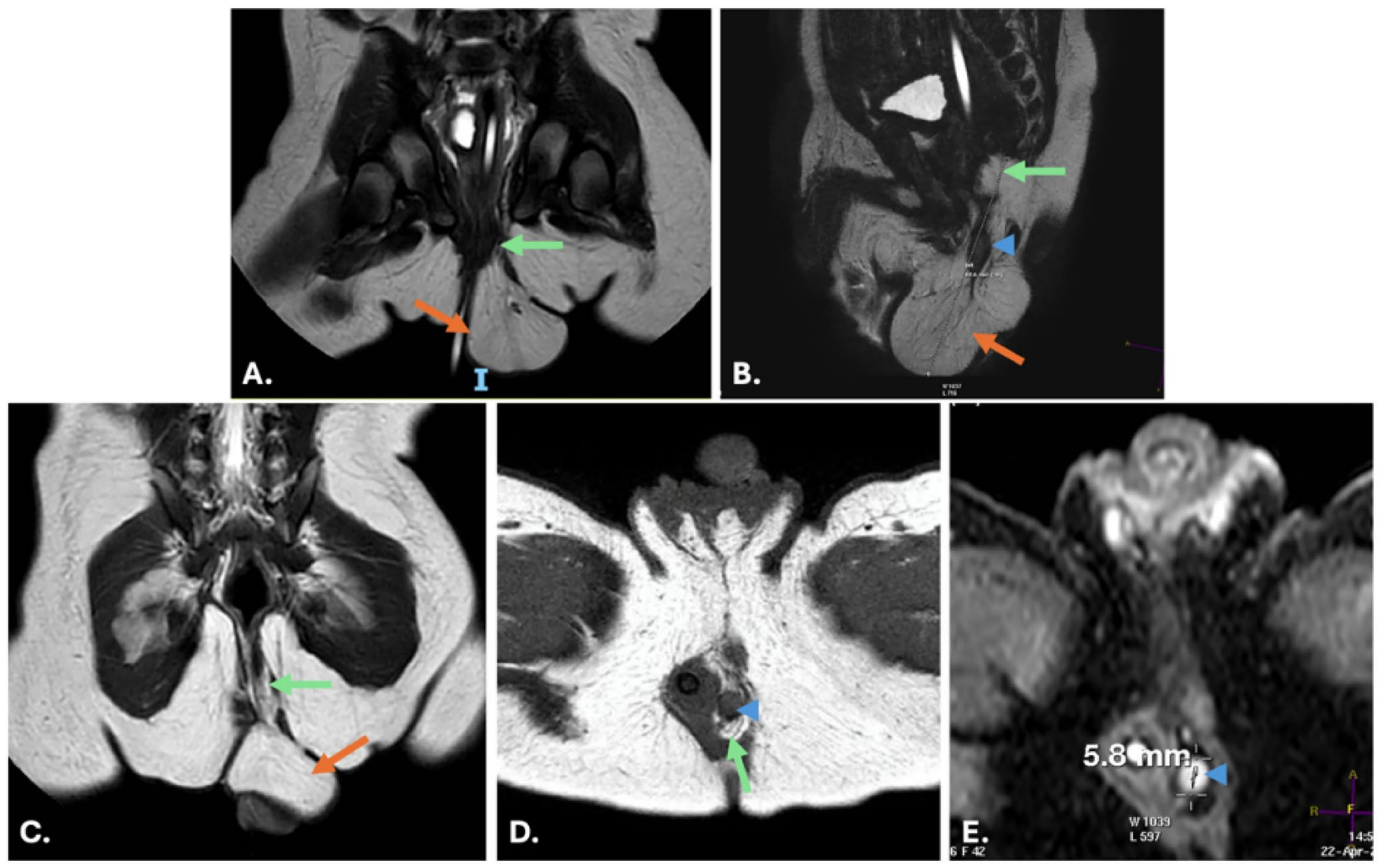
(A, B) Orthogonal planes from a T2‐weighted turbo spin‐echo sequence (A, coronal; B, sagittal). (C, D) Orthogonal planes from a T1‐weighted turbo spin‐echo sequence (C, coronal; D, axial). (E) Axial diffusion‐weighted sequence. A fat‐containing mass is located in the left posterolateral perianal region, adjacent to the anal canal. It shows a predominantly exophytic external component (orange arrow) and a smaller intrapelvic component extending between the internal and external anal sphincters (green arrow). Overall, the mass measures 6.9 × 4 × 3.3 cm (craniocaudal × anteroposterior × transverse). A subcentimeter solid nodule (arrowhead) is identified within the lesion, demonstrating facilitated diffusion (ADC 1.7) (image E). Taken together, these findings are suggestive of a type I pelvic teratoma.

Given the presence of a presacral mass and anal stenosis, Currarino's triad was considered in the differential diagnosis. However, no sacral anomalies were identified on imaging and no anorectal malformation was confirmed after surgical evaluation.

The postoperative course was uneventful. The patient is currently under follow‐up with periodic ultrasound examinations and tumor marker monitoring. AFP levels decreased to normal values after tumor resection. MRI would be considered if any suspicious findings were detected during follow‐up. At 10 months of follow‐up, the patient remains asymptomatic with normal bowel function.

This case underscores the importance of a comprehensive, multidisciplinary diagnostic approach to the evaluation of neonatal sacrococcygeal masses [1, 2]. MRI was crucial in differentiating between a benign lipoma and a type I teratoma, which, although usually benign, may entail functional and surgical risks (Figure 3) [1, 2, 3]. Assessment of the anal sphincter was also fundamental for comprehensive and timely management. Early recognition and surgical treatment are essential to reduce the risk of functional complications and potential malignant transformation.

**FIGURE 3:**
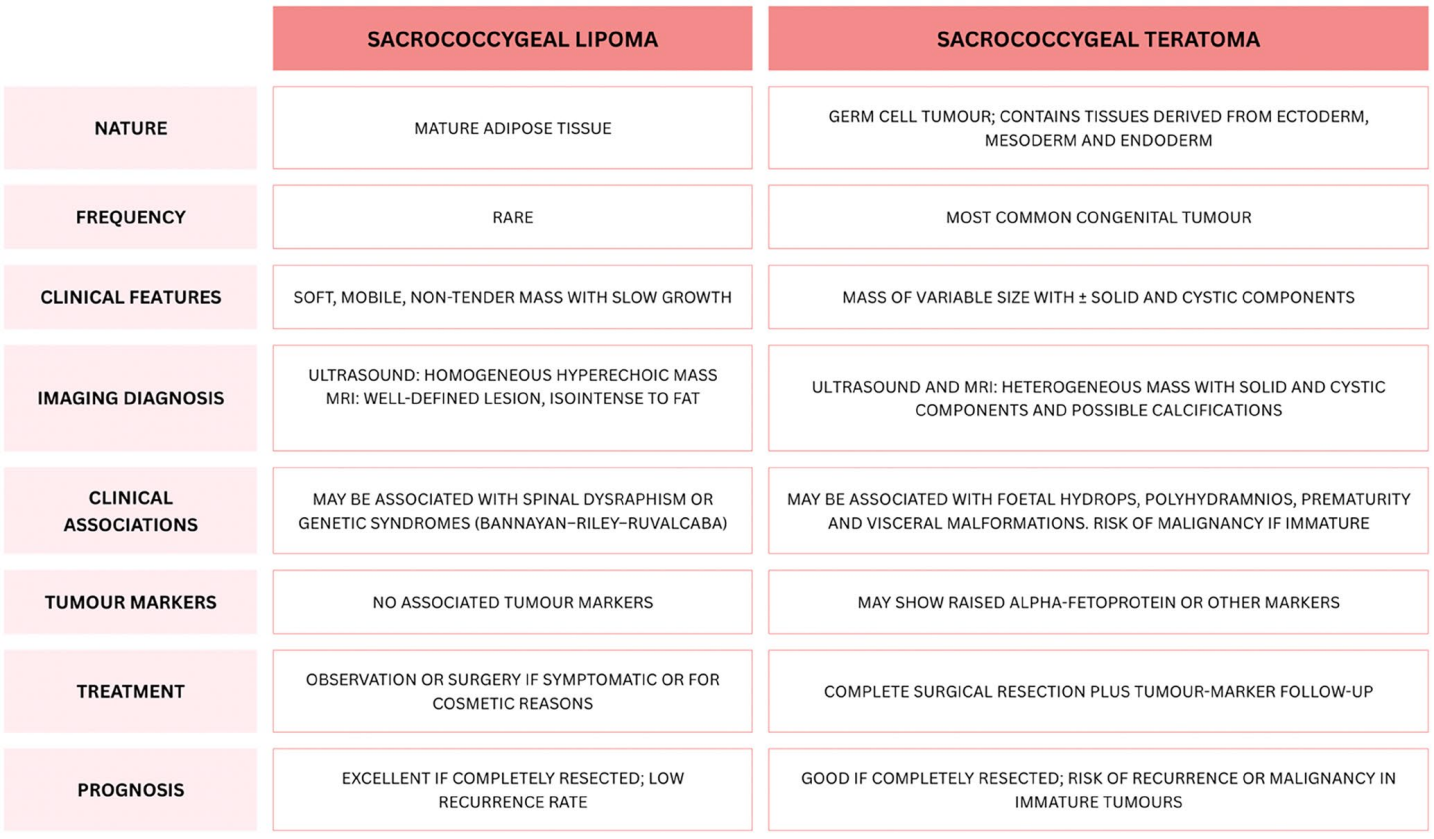
Comparison of sacrococcygeal lipoma and sacrococcygeal teratoma in neonates.

## Author Contributions

**Delia Argüelles Balas:** conceptualization, data curation, investigation, methodology, visualization, writing – original draft, writing – review and editing. **Rosa María Zubimendi Pérez:** data curation, resources, visualization, writing – original draft, writing – review and editing. **Carolina González González:** supervision, writing – original draft, writing – review and editing. **Rafael Arroyo Crespo:** writing – review and editing. **Juan López Carnero:** supervision, writing – original draft, writing – review and editing.

## Funding

The authors have nothing to report.

## Ethics Statement

Written informed consent was obtained from the patient to publish this case report in accordance with the journal's patient consent policy.

## Consent

Written informed consent was obtained from the patient's legal guardians for the publication of this case report and any accompanying images.

## Conflicts of Interest

The authors declare no conflicts of interest.

## Data Availability

Data sharing is not applicable to this article as no datasets were generated or analyzed during the current study.
